# Characterization of starch structures isolated from the grains of waxy, sweet, and hybrid sorghum (*Sorghum bicolor* L. Moench)

**DOI:** 10.3389/fnut.2022.1052285

**Published:** 2022-12-13

**Authors:** Xuemin Kang, Wentao Zhu, Tongcheng Xu, Jie Sui, Wei Gao, Zhiquan Liu, Haichun Jing, Bo Cui, Xuguang Qiao, A. M. Abd El-Aty

**Affiliations:** ^1^Department of Food Science and Engineering, Shandong Agricultural University, Taian, China; ^2^State Key Laboratory of Biobased Material and Green Papermaking, Qilu University of Technology, Shandong Academy of Sciences, Jinan, China; ^3^School of Food Science and Engineering, Qilu University of Technology, Shandong Academy of Sciences, Jinan, Shandong, China; ^4^Shandong Academy of Agricultural Science, Jinan, Shandong, China; ^5^Institute of Botany, Chinese Academy of Sciences, Beijing, China; ^6^Department of Pharmacology, Faculty of Veterinary Medicine, Cairo University, Giza, Egypt; ^7^Department of Medical Pharmacology, Medical Faculty, Ataturk University, Erzurum, Turkey

**Keywords:** inbred and hybrid sorghum starch, chain length distribution (CLD), lamellar structure, ordered structure, hydrolysis rate

## Abstract

In this study, starches were isolated from inbred (sweet and waxy) and hybrid (sweet and waxy) sorghum grains. Structural and property differences between (inbred and hybrid) sweet and waxy sorghum starches were evaluated and discussed. The intermediate fraction and amylose content present in hybrid sweet starch were lower than those in inbred sweet starch, while the opposite trend occurred with waxy starch. Furthermore, there was a higher A chain (30.93–35.73% waxy, 13.73–31.81% sweet) and lower B_2_ + B_3_ chain (18.04–16.56% waxy, 24.07–17.43% sweet) of amylopectin in hybrid sorghum starch. X-ray diffraction (XRD) and Fourier transform infrared reflection measurements affirm the relative crystalline and ordered structures of both varieties as follows: inbred waxy > hybrid waxy > hybrid sweet > inbred sweet. Small angle X-ray scattering and ^13^C CP/MAS nuclear magnetic resonance proved that the amylopectin content of waxy starch was positively correlated with lamellar ordering. In contrast, an opposite trend was observed in sweet sorghum starch due to its long B_2_ + B_3_ chain content. Furthermore, the relationship between starch granule structure and function was also concluded. These findings could provide a basic theory for the accurate application of existing sorghum varieties precisely.

## Graphical Abstract

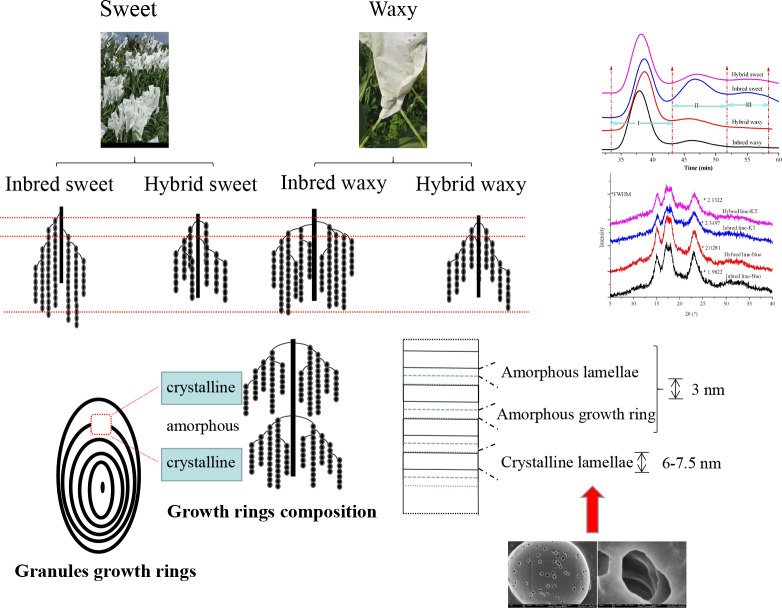

## Introduction

As the most abundant carbohydrate reserve in plants, starchy food is the staple diet of much of the world’s population. In addition to its use in food, starch has also been used in papermaking, adhesives, feed, textiles, pharmacies, and new energy due to its valuable characteristics, such as thickener, gelling agent, binder, film-formation, renewable, and biodegradable properties (1). Starch is composed of two major molecular components, amylose and amylopectin (2). Amylose, consisting of linear and rare branches, plays a critical role in the structure and properties of starch. Amylopectin is the major component of starch (90–95% for waxy and 75–80% for normal starch) and is larger than amylose (3). The branched amylopectin chains interact to form a double helix, the main component of crystallized semicrystalline layers. The alternating double helix and amorphous lamellae (amylose and branched point) bands constitute the growth rings (4). Over the past decades, research to establish the model between fine structure and properties has never been stopped: the ratio of amylose-amylopectin, molecular weight, branching degree and chain length distribution (CLD) of amylopectin, lamellar and crystalline structures of the granules have been widely investigated, which have a close impact on the application of starch (5).

Starch comes from a wide range of sources, mainly cereals, and sorghum is the top fifth providing starch, after maize, wheat, rice, and barley (6). As a climate-smart agricultural plant, sorghum (*Sorghum bicolor* L. Moench) species, which have a strong ability to tolerate drought and heat (abiotic) stresses and require minimal fertilizers, are widely planted in semiarid areas of the world (7). Characteristics such as low cost, wide source, and strong environmental adaptability indicate that sorghum has great development space for various fields. Starch is the principal component of sorghum, accounting for >80% of the total dry matter weight in the grains (8). Thus, further study on the structure of sorghum starch is of great importance in expanding its application. Sorghum starch granules appear in a mixed population of small and large sizes, particularly spherical and polygonal, with indentations and pores randomly distributed on the surface (9). The internal lamellar structure characteristics of sorghum starch extracted from sorghum flour have been investigated during water absorption at room temperature by small-angle X-ray scattering (SAXS) techniques, and it was found that the diameter of sorghum granules had a bimodal distribution and that the lamellar patterns were similar to those of other cereal starches (9). The effect of amylose content and granule morphology on sorghum starch structure and functional properties has been reported (10, 11). Brewing is one of the most critical uses of sorghum seeds in China. Li et al. (12) investigated the change in the delicate molecular structures of sorghum starch during steaming for Baijiu production. However, the shortcomings of native sorghum starch, such as retrogradation, low paste clarity, higher pH and shear sensitivity, and low thermal resistance, limit its application. Therefore, to enhance the application of sorghum starch, physicochemical, enzymatic and biotechnology methods treatments of sorghum starch were used to alter its properties (13–17). Hybrids, as the most straightforward method in biotechnology, were used to strengthen the validity of dominant traits, which was related to the dominance and overdominance hypothesis, especially for F1 (18, 19). To date, differences in the fine structure and properties of inbred and hybrid rice and maize starch have been reported (20–23). Currently, the sorghum market is minimal, mainly as a substitute for corn (24). Forage, ethanol, and edible sorghum are the three main categories in China (25). Furthermore, wide varieties are included in every category, including hybrid and inbred varieties. The plant characteristics of waxy and normal sorghum in inbred and hybrid lines have been widely studied (26–28). To accurately control the application of different types of sorghum starch, research on the structure and properties of inbred and hybrid sorghum starch, which are widely planted, is necessary.

Therefore, this study aimed to deeply analyze the relationship between fine structure and physicochemical (thermodynamics and digestibility) properties of sweet, waxy inbred, and hybrid sorghum starch, which is widely planted in China. Confocal laser scanning microscopy (CLSM) of starch growth rings, wide and small-angle X-ray scattering (XRD and SAXS) of the lamellar crystalline structure, ^13^C/MAS NMR of the superhelix structure at the granular level, and internal cluster structure of amylopectin have been exploited, which provide the necessary basic theory for the precise application of sorghum starch.

## Materials and methods

### Materials

Inbred sweet, waxy and hybrid sweet, waxy sorghum are the top two varieties planted and used as brewing and feed in China. Ten kinds of sweet sorghum and five types of waxy sorghum were selected as objects, which were kindly sponsored by the Institute of Botany, the Chinese Academy of Sciences (Dongying, China). According to the significance of the experimental results, four representative sorghum samples were selected: inbred waxy (Hunan-nuo), hybrid waxy (Liaoning-11 with parents of LA-34 and NK1), inbred sweet (Keller) and hybrid sweet (Ketian 2nd with parents of H37 and M81E). These four sorghum varieties were planted in the same experimental field in Dongying, China, in 2021. There is no genetic correlation between them, while hybrid and inbred waxy and sweet are applied to brewing, edible, and feed. Sodium hydroxide was procured from Hengxing Chemical Reagent Manufacturing Co., Ltd. (Tianjin, China). Absolute ethanol (≥99.7%) and DMSO (≥99.7%) were provided by Tianjin Fuyu Fine Chemical Co., Ltd. (Tianjin, China). Porcine pancreatic *a*-amylase (100 U/mg, CAS# 9032-08-0) and amyloglucosidase (10 × 10^4^ U/ml, CAS# 9032-08-0) were acquired from Sigma–Aldrich (St. Louis, MO, USA) and Yuanye Biotechnology (Shanghai, China), respectively. Urea, iodine and potassium iodide were provided by Sinopharm Chemical Reagent Co., Ltd. (Beijing, China). Sodium cyanotrihydridoborate and trisodium 8-aminopyrene-1,3,6-trisulfonate were acquired from Sigma–Aldrich Chemical Co., Ltd. (Auckland, New Zealand).

### Starch isolation

Sorghum grains (200 g) were mixed with 1 L 0.3% sodium hydroxide solution and placed at 4°C in the refrigerator for 24 h. After that, they were rinsed with deionized water until they became colorless and homogenized with a multifunctional cooking machine (HX-PB908, Foshan, China) at room temperature for 30 s. Afterward, the homogeneous slurry was filtered by a 200 mesh sieve (Jinyuan Colander, Hengshui, China). The precipitate of bran residue was discarded. Again, the remaining part was soaked in 1 L 0.3% sodium hydroxide solution and kept in cold storage at 4°C for 24 h. Next, the slurry was treated by centrifugation at 3,000 × g for 15 min with 200 ml 0.3% sodium hydroxide solution, 200 ml absolute ethanol, and 200 ml deionized water at 3,000 × g for 15 min successively. The brown, red, or yellow layer (containing protein and fibers) presented on the top of the precipitate layer was removed with a spoon and abandoned. Finally, the precipitated starch was washed with deionized water five times, placed in a 30°C oven for 20 h, and ground to pass through a 200 mesh sieve.

### Structural analysis

#### Starch purity and amylose content

The components of the extracted sorghum starch were detected and analyzed. The starch content was detected by a Starch Content Assay Kit (Macklin S930081-100T/EA); the protein content was detected by the Kjeldahl method, and the fat content was determined using Soxhlet extraction. These conventional experimental methods were not described in detail.

Urea-DMSO (UDMSO) solution is a mixture of 10% *(v/v)* 6 M urea and 90% (*v/v*) dimethyl sulfoxide. The iodine solution was made by dissolving 1 g I_2_ and 10 g KI in 500 ml of deionized water. Twenty milligrams of accurately weighed starch was dissolved into 8 ml of UDMSO solution and vortexed for 2 min. Next, the solution was heated in a water bath for 30 min at 85°C. After the temperature was cooled to 25°C, the solution was adjusted to a volume of 25 ml with purified water. Then, 3 ml of diluted solution with a pipette gun was mixed with 1 ml of iodine solution, and the volume was adjusted to 50 ml with purified water. Finally, the absorbance of the sample was logged at 630 nm. The standard curve y = 0.0133*a* + 0.0413 with *R*^2^ = 0.9993 was prepared with a minor modification of (29).

#### Chain length distribution of amylopectin

The CLD of different varieties of sorghum starch was analyzed by high-performance anion-exchange chromatography (HPAEC) (Thermo Fisher, DIONEX™ ICS-5000^+^ Waltham, MA, USA) as described by Li et al. (30) with minor modifications. Starch powder (20 mg) was added to 4 ml 90% (*v/v*) DMSO and heated for 20 min at 100°C with continuous stirring at 10 rpm. Then, 4 ml of absolute ethanol was added and mixed evenly. After centrifugation at 3,500 × g for 15 min, the precipitate was mixed with 4 ml 50 mM sodium acetate buffer solution (pH 4.0) and heated at 100°C for another 20 min with continuous stirring at 10 rpm. Then, 10 μl isoamylase (180 U/ml, Megazyme, Bray, Business Park, Ireland) was dispersed after the starch suspension equilibrium at 40°C for 10 min and kept at 40°C for 1 day with continuous stirring at 10 rpm. Finally, the suspension was sterilized in a boiling water bath at 100°C for 10 min. Then, 85% solution A (150 mM NaOH) and 15% solution B (150 mM NaOH and 500 mM CH_3_COONa) were used as the mobile phase, with a flow rate of 0.5 ml/min through the CarboPac PA20 column, and the branched chain of amylopectin was eluted.

#### Molecular weight distribution

The molecular weight distribution of sorghum starch was measured by size-exclusion chromatography with multiangle light scattering (SEC-MALLS) paired with refractive index (RI) detection, as described by Lin et al. (20). A 5 mg starch sample was dispersed into 5 ml DMSO (0.5% LiBr) and heated at 80°C for 3 h. Determination conditions: gel exclusion chromatography (Ohpak SB-805 HQ (300) × 8 mm); column temperature: 60°C; injection volume: 100 μl; and mobile phase (5 ml DMSO, 0.5% LiBr).

#### Microstructure of starch samples

##### Maltese cross

Maltese crosses of sorghum starches were observed by a polarizing light microscope 200 × (Olympus, DP73, UHGLGPS, Tokyo, Japan).

##### Scanning electron microscopy

The morphology of starch granules was investigated by scanning electron microscopy (SEM) (Hitachi regulus 8220, Tokyo, Japan) (31).

##### Confocal laser scanning microscopy

Solution A was a mixture of 50% *(v/v)* glycerol and 50% (*v/v*) deionized water; 13% (*w/w*) deionized water, 85% (*w/w*) glycerol, and 2% (*w/w*) agar were mixed and heated at 100°C for 5 min, called Solution B. Starch samples (10 mg) were mixed with 1 M sodium cyanoborohydride aqueous solution (15 μl) and 6 mM APTS acetic acid solution (15 μl). Next, the mixtures were placed at 30°C for 8 h and washed with deionized water five times. Then, 100 μl A solution and 400 μl B solution (30°C) were quickly added to the starch samples and mixed vigorously for 2 min. The starch samples were observed by a laser confocal microscope (Olympus, FV1200, Tokyo, Japan).

#### X-ray diffraction and small-angle X-ray scattering

The crystallinity of dried powder (200 mesh sieve) of different sorghum starches was determined by an X-ray diffractometer with a diffraction angle of 2θ = 4^°^–40^°^ (Ultima IV, Kuraray Co., Ltd., Tokyo, Japan) at a target voltage and current of 40 kV and 40 mA, respectively (32).

One gram of sorghum starch was blended with 3 ml of hydrated-starch deionized water and kept for 72 h at 30°C (vortex mixed every 10 h). Then, the hydrated starch was determined by a Bruker NANOSTAR SAXS system of Hangzhou Yanqu Information Technology Co., Ltd., applying three pinhole collimation for focusing, Cu Kα radiation with purity >99.5%, and a VANTEC500 2D detector (resolution 68 μm _×_ 68 μm). The automatic sampler was used under vacuum (<0.1 mbar) with *q* in the range of 0.07–2.1 nm.

#### Fourier transform infrared spectroscopy and solid-state NMR carbon spectroscopy (^13^C NMR) analysis

A NICOLET iS10 Thermo Fisher Scientific (Massachusetts, USA) was used to determine the Fourier transform infrared spectroscopy (FTIR) spectra of different varieties of sorghum starch. A 1 mg starch powder was mixed with 100 mg KBr, and a thin sheet was acquired by pressing at a pressure of 12 MPa for 40 s.

Solid-state NMR spectroscopy was performed by Bruker BioSpin GmbH (Bruker Instrument, Inc., Billerica, MA, USA). The ^13^C CP/MAS NMR spectra were implemented with a MAS spin rate of 10 kHz. The recovery time was 4 s, the number of scanning accumulations was 1,024, the acquisition time was 0.01 s, and the spectral width was 50505.1 Hz.

### Physicochemical properties

#### Thermal analysis

A DSC-200FC differential scanning calorimeter (NETZSCH, Selb, Germany) was used to determine sorghum starch thermal properties. Three milligrams of starch powder and 9 μl of distilled water were mixed in an aluminum crucible and sealed with a lid. After 12 h of equilibrium, the prepared crucibles were heated from 40 to 140°C within 10°C/min.

#### *In vitro* digestion

The *in vitro* digestibility of the three different varieties of sorghum starch was analyzed as described by Englyst and Hudson (33). Sorghum starch powder (40 mg) was added to pH 5.2 acetate buffer (35 ml). Then, the suspension was mixed with a 10 ml enzyme solution, which consisted of amyloglucosidase (30 U/ml) and α-amylase (290 U/ml) in pH 5.2 acetate buffer. Furthermore, 20 μl of starch hydrolysis solution at 20, 40, 60, 90, 120, and 180 min was removed and injected into a biosensor (Shandong Academy of Sciences, Jinan, Shandong), used to calculate the glucose content automatically. Then, the hydrolysis rate of starch was calculated according to the following formula:


$$d=\frac{a\times 45\times 0.9}{100\times 40}$$

where d is the hydrolysis rate of the starch sample at different times (%), and a is the glucose content at a different time (mg/dL).

### Statistical analyses

The data are presented as the mean values ± standard deviations. ANOVA was performed using SPSS Statistics 19.0, and *P* < 0.05 was considered statistically significant.

## Results and discussion

### Structural analysis

#### Starch purity and amylose content

The starch contents of inbred sweet, inbred waxy, hybrid sweet and hybrid waxy were 94.7, 95.7, 96.9, and 96.0%, respectively. The minor components are protein and lipid, less than 0.51 and 1.2%, respectively.

Significant differences (*P* < 0.05) in amylose contents for inbred waxy, hybrid waxy, inbred sweet, and hybrid sweet are shown in Table 1. Higher amylose content was observed in sweet sorghum than in waxy varieties.

**TABLE 1:** Parameters of amylose, molecular weight, and chain length distribution of inbred and hybrid sorghum starch.

Samples	Amylose content (%)	Mn (10^4^ kDa)	Mw (10^4^ kDa)	Dp (Mw/Mn)	Rz (nm)	DP 6–12	DP 13–24	DP 25–36	DP > 37	Average chain length (ACL)
Hybrid sweet	23.43 ± 1.41^a^	1.77 ± 0.08^a^	7.86 ± 0.01^a^	4.43 ± 0.02^a^	171.20 ± 0.74^b^	31.81 ± 0.12^b^	50.76 ± 0.38^a^	11.42 ± 0.41^a^	6.01 ± 0.11^a^	17.55 ± 0.52^a^
Inbred sweet	26.52 ± 0.90^b^	6.44 ± 0.07^b^	30.08 ± 0.01^b^	4.67 ± 0.01^b^	146.70 ± 0.86^a^	13.73 ± 0.41^a^	60.36 ± 0.13^b^	16.96 ± 0.22^b^	7.11 ± 0.07^b^	20.39 ± 0.83^b^
Hybrid waxy	6.36 ± 0.06^b^	4.06 ± 0.07^a^	7.29 ± 0.01^a^	1.80 ± 0.02^a^	197.50 ± 0.73^a^	35.73 ± 0.39^b^	47.74 ± 0.20^a^	11.18 ± 0.17^a^	5.38 ± 0.14^a^	16.87 ± 0.69^a^
Inbred waxy	5.65 ± 0.08^a^	5.10 ± 0.07^b^	11.84 ± 0.01^b^	2.32 ± 0.01^b^	212.70 ± 0.97^b^	30.93 ± 0.61^a^	51.03 ± 0.11^b^	11.81 ± 0.25^b^	6.23 ± 0.05^b^	18.98 ± 0.48^b^

Values are represented as the mean ± SD; values in the column with different superscript letters are significantly different at *P* < 0.05.

#### Molecular weight distribution

**FIGURE 1:**
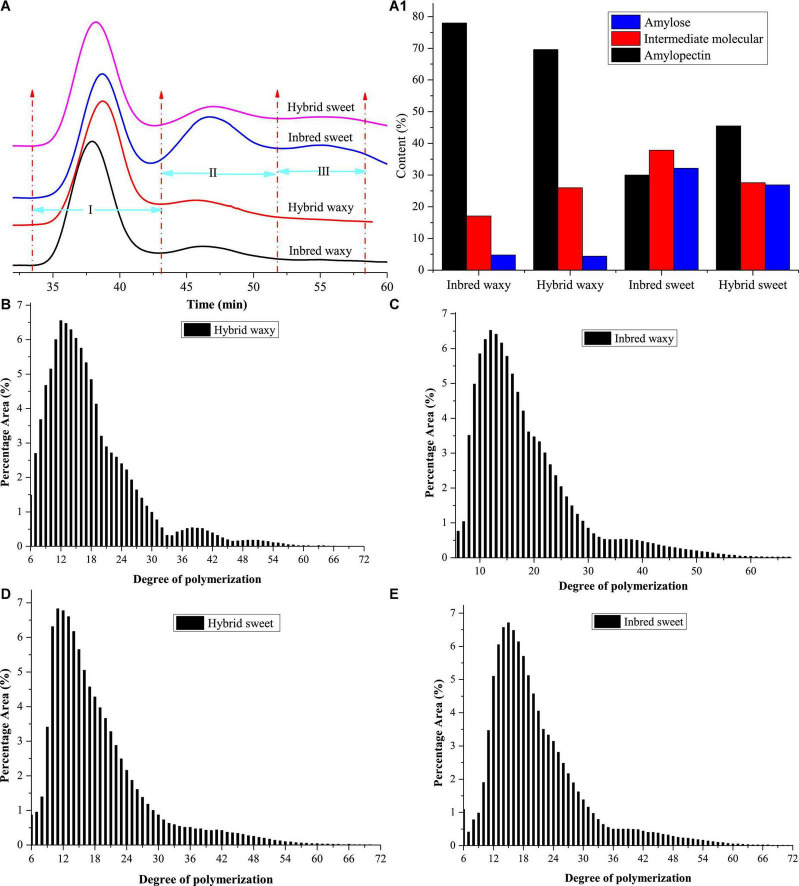
Molecular weight and chain length distribution of starch from waxy, sweet, and hybrid sorghum. **(A,A1)** Molecular weight distribution; **(B–E)** chain length distribution.

The molecular weight distributions of hybrid and inbred waxy and sweet sorghum starch are shown in Figures 1A,A1 and Table 1. Three fractions, amylopectin (I), intermediate molecular (II), and amylose (III), were observed depending on the time sequence of occurrence. A larger proportion of the II and III fractions was observed in sweet sorghum varieties than in waxy varieties. Furthermore, the maximum proportion of fractions II and III in inbred sweet and the minor proportion of fractions II and III in inbred waxy were consistent with the determination result of amylose content in inbred waxy and sweet sorghum starch. Furthermore, a significantly lower fraction I and higher fraction II could be observed in hybrid waxy starch. At the same time, there was less than 1% amylose in inbred waxy starch than in hybrid waxy starch, which means that part of fraction II could interact with iodine, which increased the amylose content. However, this phenomenon was not observed in sweet sorghum starch. Simultaneously, Dp and Rz values were also calculated, as shown in Table 1. Dp indicates the molecular distribution of starch, and a larger Dp value indicates a wider molecular distribution. In contrast, the closer the value is to 1, the more uniform the dispersion is. These results showed that waxy starch (*Dp* = 1.80 and 2.32) was lower than sweet varieties (*Dp* = 4.43 and 4.76), which means a narrower molecular weight distribution. Better homogeneity could be observed in hybrid varieties (1.80 < 2.32; 4.43 < 4.76), which would be related to the chain length distribution. In addition, the branching degree of starch could be determined by the Rz value. The changes in the Rz value represented in Table 1 (146.7–171.2; 212.7–197.5) showed that the branching degree of sweet starch and waxy starch was opposite in the hybrid and inbred varieties.

#### Amylopectin chain length distribution

Amylopectin consists of three types of branched chains. A chain connected to C or B chains through α-D-(1,6)-glucosyl linkages has no branches, and its degree of polymerization (DP) is 6–12. B chain, connected to other B chains or C chains through α-D-(1, 6)-glucosyl linkage, B_3_ chain DP > 37, B_2_ chain 25–36, and B_1_ chain 13–24. C-chain, only one per amylopectin with a reducing end (34). The CLDs of the hybrid and inbred waxy and sweet sorghum starch are shown in Figures 1B–E, and their amounts are reflected in Table 1. The average chain length (ACL) of hybrid sweet starch-17.55% and inbred sweet starch-20.39% was obviously higher than that of hybrid waxy starch-16.87 and inbred waxy starch-18.98%, which was consistent with the Dp value of starch. Furthermore, the CLD and ACL of amylopectin also presented distinctions among hybrid and inbred waxy sorghum starch, and a higher Dp value appeared with higher ACL. Compared with inbred sweet and waxy starch, significantly higher A- and lower B-chains were observed in hybrid starch. In addition, the A chain content of inbred sweet starch (31.81%) was negatively correlated with the intermediate fraction and amylose content of hybrid sweet starch (23.43%) with *P* < 0.05. Compared with hybrid sweet starch, a lower A chain (13.73%) and a higher B chain (60.36%) were observed in inbred sweet starch. For the waxy sample, the higher A chain and lower B_1_ chain were only positive for the intermediate fraction and amylopectin, which have no relationship with amylose content. The change in the A and B_1_ chains of inbred and hybrid waxy sorghum was similar to that of sweet sorghum starch. These results were the basis for analyzing the fine structure of starch granules.

#### Microstructure of starch samples

**FIGURE 2:**
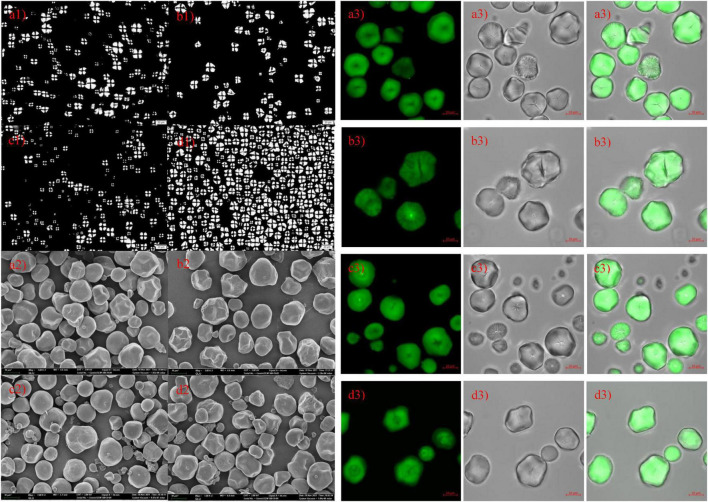
Polarizing, scanning electron microscopy and confocal laser scanning microscopy of starch from waxy, sweet, and hybrid sorghum. **(a1–a3)** Hybrid waxy starch; **(b1–b3)** inbred waxy starch; **(c1–c3)** hybrid sweet starch; **(d1–d3)** inbred sweet starch.

Microscope, SEM, and CLSM photographs of hybrid and inbred waxy and sweet sorghum starch are shown in Figures 2a–d,a1–d1,a2–d2, respectively, a: hybrid waxy starch; b: inbred waxy starch; c: hybrid sweet starch; and d: inbred sweet starch. Figures 2a1–d1 shows that the maltese cross was observed in all sorghum starch samples with different sizes and shapes, especially the starch of hybrid sweet starch. Three or four types of particle sizes in various shapes can be observed in Figures 2a2–d2. Large and small sorghum starch granules were polygonal and round, with micropores widely distributed on the surface. More small granules could be observed in sweet samples than in waxy starch. Additionally, more micropores were concentrated in the dents of the waxy sample, which was magnified 5,000 times (not shown in the figures). Moreover, the micropores on the surface of starch granules were empty channels connecting the surface to the hilum, which can be observed by CLSM in Figures 2a3–d3. The molecular fluorescent probe APTS can specifically react to the reducing end of starch molecules and effectively label starch particles (35). Amylose molecules contain more reducing ends per unit of glucose residue than amylopectin molecules; thus, amylose in starch particles can be strongly labeled. Channels are the pathways of related enzymes in the process of starch synthesis. It is also a channel for hydrolytic solvents or enzymes entering. Interestingly, the micropores on the small starch granules are denser than those on the large ones, and the reason is unclear. The internal structure-growth ring of starch is shown in Figures 2a3–d3, also called crystalline and amorphous layers in starch granules (36). The lighter color in Figures 2c3,d3 represents a higher amylose content in sweet sorghum starch, also called the amorphous growth ring. In addition, a looser periphery was observed in inbred and hybrid waxy starch, which was consistent with a previous report stating that waxy starch with little amylose lacks the winding of amylopectin and amylose (37). Furthermore, a partial branch point of sorghum amylopectin would make up the amorphous lamellar. Combined with the amorphous growth ring, a higher amylopectin content caused a thick amorphous structure. Compared with hybrid sweet starch, the peripheral growth ring of inbred sweet starch is not close-packed, which was attributed to the higher sum of the B_2_ + B_3_ chains of amylopectin. In conclusion, there was no apparent difference in the granule morphology between hybrid and inbred waxy and sweet sorghum.

#### X-ray diffraction and small-angle X-ray scattering

**FIGURE 3:**
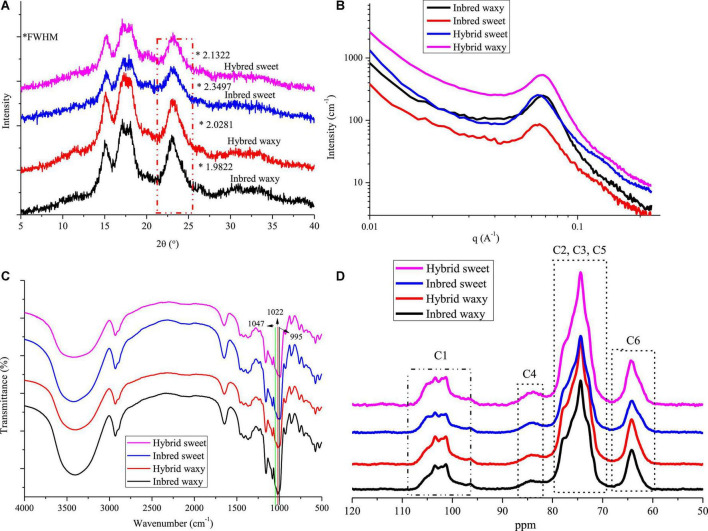
X-ray diffraction (XRD), small-angle X-ray scattering (SAXS), FTIR, and ^13^C CP/MAS NMR of starch from waxy, sweet, and hybrid sorghum. **(A)** XRD; **(B)** SAXS; **(C)** FTIR; and **(D)** ^13^C CP/MAS NMR.

The XRD patterns of hybrid and inbred waxy and sweet sorghum starches are presented in Figure 3A. These four types of sorghum starches exhibited typical A-type XRD patterns with clear signature doublet peaks at 2θ = 17° and 18°, except for the strong reflections at 2θ = 15.1° and 23°, which proved that there was no difference in the crystalline type of sorghum starch between hybrid and inbred varieties (38). Significant differences (*P* < 0.05) in the full width at half maxima (fwhm) among sorghum starches were observed, being 2.1322 (hybrid sweet), 2.3497 (inbred sweet), 2.0281 (hybrid waxy), and 1.9822 (inbred waxy). The fwhm value of these four starches was opposite to the amylopectin content. This phenomenon was determined by the differences in the content of amylopectin, which would form a double helix structure between the adjacent side chain of amylopectin, constituting the crystalline region of starch particles (2). In contrast, linear amylose and amylopectin branched points constitute the amorphous region of starch particles. The lower fhwm value of waxy samples proved that starch granules with higher amylopectin content have a higher semicrystalline structure (39). Furthermore, a higher A chain in hybrid waxy starch (35.73%) than inbred Nuo starch (30.93%) was positively correlated with the fhwm value, indicating that short A-chains in waxy starch do not readily participate in the formation of the semicrystalline layer. The opposite result was noticed in sweet sorghum starch due to the higher amylose content (26.52%) in inbred sweet than in hybrid sweet.

The SAXS of hydrated starch was determined in this experiment, and only hydrated starch is shown in Figure 3B. The SAXS technique has been widely used to reflect the internal structure of starch granules (alternating amorphous and semicrystalline). A broad scattering peak in the range of 0.04 < *q* < 0.09 A^–1^ is shown in Figure 3B, Revealing an average total thickness between crystalline and amorphous regions in the growth ring of inbred waxy (0.9194), hybrid waxy (0.9194), inbred sweet (1.003), and hybrid sweet (0.9808). The higher growth thickness of inbred sweet than hybrid sweet could be attributed to the higher B_2_ + B_3_ content, whose branched points distributed in the amorphous lamella increased the growth thickness. Furthermore, the fwhm of SAXS could reflect the degree of peak intensity, and it decreased in the following order: inbred waxy < hybrid waxy < hybrid sweet < inbred sweet. This result proved that hybrid waxy sorghum starch had a perfect crystalline layer compared to the inbred waxy one. The opposite result was noted in sweet sorghum starch. The highest fwhm of inbred sweet starch may be related to the highest sum of the B_2_ + B_3_ chain distribution, which increased the thickness of growth rings and reduced the compactness of the crystal structure. Furthermore, the relative area of the scattering peak increased in the following order: inbred waxy > hybrid waxy > hybrid sweet > inbred sweet, reflecting the content of the double helix structure. A higher relative area indicates more helix content. This phenomenon could not only be ascribed to the higher amylopectin content but also related to the higher B_2_ + B_3_ content, consistent with the XRD results.

#### Fourier transform infrared spectroscopy and solid-state ^13^C CP/MAS NMR

Fourier transform infrared spectroscopy spectra are used to record the structural differences of starch granules. Characteristic peak absorption bands representing OH, C-H bonds, –CH_2_ bending and –COO stretching, and C-H bending appeared at 3,245 cm^–1^, 2,924 cm^–1^, 1,343 cm^–1^, and 1,000 cm^–1^ and are shown in Figure 3C (40). Similar FTIR peaks were observed in these four sorghum samples; however, the peak intensity at approximately 1,000 cm^–1^ was different in all samples. The bands at 1,047 (995) and 1,022 cm^–1^ are susceptible to crystalline starch transformation and amorphous structure, respectively (41, 42). A decreased ratio of 1,047/1,022 cm^–1^ was observed in the order of inbred waxy < hybrid waxy < hybrid sweet < inbred sweet, which exhibited a similar trend with the fwhm of SAXS. In addition, the opposite order was observed in the ratio of 1,022/995 cm^–1^, which indicates the highest relative crystallinity of inbred waxy starch. This result was similar to XRD and SAXS; starch with higher amylopectin content had more crystalline structure due to more helix structure in semicrystalline regions. A higher A chain and the sum of B_2_ + B_3_ played a vital role in forming a loose structure, as illustrated by inbred sweet starch.

^13^C CP/MAS spectra of all sorghum starches are displayed in Figure 3D. Resonances that appeared at 97–104, 57–64, and 79–84 are C_1_, C_6_, and C_4_ in hexopyranoses of starch, respectively (43). At approximately 69–74 ppm, the overlapping resonances are assigned to C_2_, C_3_, and C_5_ (44). The C_1_ resonances of sorghum starch were triplets, which is a typical A-type crystalline characteristic. The relative area of C_1_ resonances progressively decreased in the order hybrid waxy < inbred waxy and inbred sweet < hybrid sweet, denoting that there were more ordered crystalline domains in inbred waxy than hybrid waxy, in contrast to hybrid sweet. This result is consistent with SAXS.

Based on XRD, SAXS, FTIR, and NMR, the differences in the structure between hybrid and inbred waxy and sweet sorghum starch granules should not be ignored. For sweet sorghum varieties, a decrease in amylose and the intermediate fraction and increased short-chain content were observed in the hybrid sweet sample compared with inbred sweet. However, the opposite behavior was observed in waxy sorghum starch. Amylopectin content could be the main factor of crystal order in waxy sorghum starch, while for sweet sorghum starch, the B_1_ + B_2_ chain content had the maximum impact. In short, the characteristics of starch were determined by genetic material, which played a decisive role in the internal structure of starch granules, the double helix structure, crystal structure and order.

### Thermal properties and *in vitro* digestion

**FIGURE 4:**
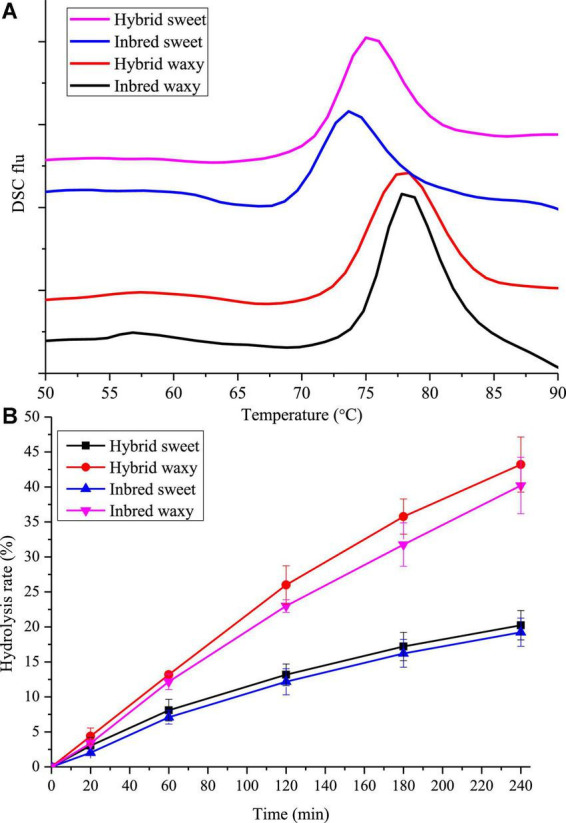
Thermodynamics and *in vitro* digestion of starch from waxy, sweet, and hybrid sorghum. **(A)** DSC; **(B)** *In vitro* digestion.

The gelatinization temperatures T_o_, T_p_, T_c,_ and gelatinization enthalpy (ΔH) for hybrid and inbred waxy and sweet sorghum starch are displayed in Figure 4A and Table 2. According to previous studies, the transition temperature (T_p_) is considered to be a microcrystalline quality index related to the orderly structure of the amylopectin double helix, which provides structural stability and renders the granules more resistant to gelatinization, and ΔH is a measure of the content loss of molecular order (45, 46). Significant diversity could be observed in Table 2, and the T_p_ ranged from 75 to 78°C. The order of T_p_ increased in the following order: inbred waxy > hybrid waxy > hybrid sweet > inbred sweet, which was similar to the results of XRD and SAXS. This phenomenon illustrated that the double-helical structure of inbred sweet starch was the loosest (47). The ΔH values, which have a consistent trend with T_p_, illustrate a more crystalline structure in inbred waxy, consistent with ^13^C CP/MAS and FTIR.

**TABLE 2:** Parameters of DSC, SAXS, and FTIR of starch from waxy, sweet, and hybrid sorghum.

Sample	T_o_ (°C)	T_p_ (°C)	T_c_ (°C)	ΔH (J/g)	Area	Fwhm	1,047/1,022 cm^–1^	1,022/995 cm^–1^
Hybrid sweet	73.95 ± 0.64^b^	77.25 ± 0.07^a^	80.30 ± 0.85^b^	14.53 ± 0.63^b^	4.28 ± 0.10^a^	0.019 ± 3^∧^10**^–^**^3a^	1.09 ± 0.04^b^	1.01 ± 3^∧^10**^–^**^3a^
Inbred sweet	71.05 ± 0.07^a^	75.45 ± 0.21^a^	80.01 ± 0.14^b^	11.37 ± 0.61^a^	4.01 ± 0.23^a^	0.029 ± 1^∧^10**^–^**^3b^	1.06 ± 0.02^a^	1.02 ± 3^∧^10**^–^**^3b^
Hybrid waxy	73.90 ± 0.20^a^	78.30 ± 0.17^a^	82.86 ± 0.38^b^	17.06 ± 0.61^c^	6.54 ± 0.29^a^	0.018 ± 1^∧^10**^–^**^3a^	1.06 ± 0.01^a^	0.99 ± 2^∧^10**^–^**^3b^
Inbred waxy	74.67 ± 0.78^b^	78.73 ± 0.51^b^	83.77 ± 1.00^b^	18.47 ± 1.11^a^	8.45 ± 0.31^b^	0.017 ± 1^∧^10**^–^**^3a^	1.10 ± 0.02^b^	0.96 ± 6^∧^10**^–^**^3a^

Values are represented as the mean ± SD; values in the column with different superscript letters are significantly different at *P* < 0.05. Abbreviations in Tables 1, 2 are explained in the text.

The *in vitro* hydrolysis characteristics of all four sorghum starches are presented in Figure 4B. Nearly linear digestion curves in the first 60 min could be observed. With increasing hydrolysis time, the growth rate of the hydrolysis rate gradually slowed. The hydrolysis rate performed by waxy varieties increased from 21 to 40% during 120–240 min, more than twice that of sweet varieties (10–18%). This result could be attributed to two points: the first one is that there were more micropores on waxy starch granules than on sweet starch granules, where enzymes entered easily; the second one is that higher amylopectin content was easily broken by *a*-pancreatic amylase and glycosidase due to the existence of amylose causing the lamellar structure of starch particles to be looser, where they easily contacted hydrolase (21). Thus, the digestibility of waxy starch used in wine-making is also suitable for feed that increases livestock’s energy intake and promotes growth. In addition, compared with hybrid sweet starch with 31.81% A chain content, a higher digestion rate was observed in inbred sweet starch with 13.73% A chain content. This phenomenon proved that the digestibility of starch was remarkably actively correlated with amylopectin branch chains of DP 6–12 (48). However, the results opposite to hydrolysis of sweet samples were observed in intermediate fractions, B-chain (47.74–51.03%) and ACL (16.87–18.98%) content, which illustrated that starch granules with more intermediate fractions and B-chain have a perfect crystalline structure that prevents the entry of enzymes (49).

## Conclusion

Waxy, sweet inbred, and hybrid sorghum starch contained three parts: amylose, an intermediate fraction, and amylopectin. The inbred waxy sorghum starch had distinctively higher amylopectin, intermediate fraction, long branch chains of amylopectin, and ACL than the hybrid waxy starch. The perfect crystalline structure of inbred waxy sorghum starch was concluded by XRD, SAXS, and ^13^C CP/MAS NMR, which was also reflected by a higher ΔH and lower hydrolysis than hybrid waxy starch. In addition, almost the opposite result occurred in sweet sorghum varieties: higher amylose, intermediate fraction and lower amylopectin content were observed in sweet inbred sorghum starches than in the hybrid sample. However, the higher amylopectin long branch-chains (B_2_ + B_3_) led to a looser crystalline structure than the hybrid structure, which was reflected by the lower T_p_ and ΔH in the DSC results. This research could provide considerable information to better understand the relations between sorghum starch structures and function and offer a theoretical basis for the precise application of sorghum starch.

## Data availability statement

The original contributions presented in the study are included in the article/supplementary material, further inquiries can be directed to the corresponding author/s.

## Author contributions

XK: methodology, investigation, and writing—original draft. WZ, JS, TX, and WG: investigation. ZL and HJ: visualization. BC, XQ and AMA: formal analysis, validation, supervision, visualization, and writing—review and editing. All authors contributed to the article and approved the submitted version.
